# Elexacaftor/Tezacaftor/Ivacaftor Supports Treatment for CF with ΔI1023-V1024-CFTR

**DOI:** 10.3390/ijms26115306

**Published:** 2025-05-31

**Authors:** Yunjie Huang, Jorge Moises Gonzales Cordova, Sarah Penrod, Lisa Lynn Bendy, Pi Chun Cheng, Don B. Sanders, Michael Denning Davis, Benjamin Gaston, James Francis Chmiel

**Affiliations:** 1 Division of Pediatric Pulmonology, Allergy, and Sleep Medicine, Department of Pediatrics, Indiana University School of Medicine, Indianapolis, IN 46202, USA; jogonza@iu.edu (J.M.G.C.); sapenr@iu.edu (S.P.); lbendy@iu.edu (L.L.B.); chengp@iu.edu (P.C.C.); dbsand@iu.edu (D.B.S.); mdd1@iu.edu (M.D.D.); begaston@iu.edu (B.G.); jfchmiel@iu.edu (J.F.C.); 2 Herman B Wells Center for Pediatric Research, Indiana University School of Medicine, Indianapolis, IN 46202, USA

**Keywords:** Cystic Fibrosis (CF), CFTR, I1023-V1024, tTheratyping, CFTR modulators, nasal epithelial cells

## Abstract

Cystic Fibrosis (CF) is a common genetic disease in the United States, resulting from mutations in the *Cystic Fibrosis transmembrane conductance regulator (cftr)* gene. CFTR modulators, particularly Elexacaftor/Tezacaftor/Ivacaftor (ETI), have significantly improved clinical outcomes for patients with CF. However, many CFTR mutations are not eligible for CFTR modulator therapy due to their rarity. In this study, we report that a patient carrying rare complex CFTR mutations, c.1680-877G>T and c.3067_3072delATAGTG, showed positive clinical outcomes after ETI treatment. We demonstrate that ETI was able to increase the expression of CFTR harboring c.3067_3072delATAGTG in a heterologous system. Importantly, patient-derived nasal epithelial cells in an air–liquid interface (ALI) culture showed improved CFTR function following ETI treatment. These findings supported the initiation of ETI with the patient. Retrospective studies have suggested that the patient has shown small but steady improvement over the past two years in several clinical metrics, including lung function, body mass index (BMI), and sweat chloride levels. Our studies suggest that ETI could be beneficial for patients carrying c.3067_3072delATAGTG.

## 1. Introduction

Cystic Fibrosis (CF), caused by mutations in the *CF transmembrane conductance regulator (cftr)* gene, affects approximately one in 3,400 newborns of Caucasian descent in the US. CFTR modulators have shown significant benefits in treating patients with CF (pwCF) who carry eligible CFTR mutations, including the deletion of Phenylalanine at position 508 (ΔF508), which is the most common mutation in pwCF. However, many patients do not have access to these modulator therapies due to the rare CFTR mutations they carry.

Theratyping studies, using either a heterologous system or patient-derived samples, have been recognized as a critical path to access CFTR modulators for rare CFTR mutations [1]. Since the US Food and Drug Administration (FDA) endorsed the use of in vitro preclinical studies to expand CFTR modulator labeling for the treatment of CF in 2017, many rare CFTR variants have been suggested or approved for CFTR modulator treatment through therapying studies [2,3,4,5].

The CFTR gene with the c.3067_3072delATAGTG mutation is a rare in-frame deletion variant that translates into the ΔI1023_V1024-CFTR protein. This variant has been previously characterized in a heterologous model system, human embryonic kidney 293 (HEK293) cells, and was suggested to have defects in protein maturation and function [6]. Individuals with CF who have compound heterozygous CF-causing mutations with ΔI1023_V1024-CFTR exhibit classic CF symptoms, such as pancreatic insufficiency, a reduction in percentage predicted forced expiratory volume in 1 s (ppFEV1), and increased sweat chloride concentrations [7]. Interestingly, ΔI1023_V1024-CFTR has shown a response to Elexacaftor/Tezacaftor/Ivacaftor (ETI) in vitro [6], indicating a potential clinical benefit of ETI for people with CF (pwCF) who have ΔI1023_V1024-CFTR. However, no such cases have been reported in a clinical setting.

In the present study, we reported on a patient who carries two CF-causing mutations, c.1680-877G>T and c.3067_3072delATAGTG. Neither mutation is considered responsive to CFTR modulators. In this report, theratyping studies suggested that ETI improved ΔI1023_V1024-CFTR expression in HEK293 cells and CFTR function in the patient-derived nasal epithelial cells (NECs). Importantly, the patient showed a response to ETI treatment, supporting the use of ETI in patients carrying ΔI1023_V1024-CFTR.

## 2. Case Presentation

### 2.1. Clinical Features Before ETI

This is a 19-year-old male patient who possesses two CFTR mutations, c.1680-877G>T and c.3067_3072delATAGTG, confirmed by genetic testing. The mutation c.1680-877G>T, also known as c.1679+1643G>T and 1811+1643G>T, is a rare CFTR variant located between exons 12 and 13 of the *CFTR* gene. A computational model suggests that this mutation creates a deep intronic cryptic donor, resulting in a 53 bp pseudoexon in the final processed mRNA [8]. Importantly, a minigene containing this mutation failed to express CFTR protein in HEK293 cells [8]. Therefore, patients with this mutation typically exhibit CF symptoms when the other allele also carries a CF-causing mutation [8,9,10]. In contrast, the mutation c.3067_3072delATAGTG, another rare CFTR variant, located in exon 17a of the *CFTR* gene and is predicted to produce an in-frame deletion protein, ΔI1023_V1024-CFTR. In vitro analysis of this mutation using HEK293 cells suggests that ΔI1023_V1024-CFTR behaves similarly to a class II mutation with trafficking and conductance defects, like ΔF508-CFTR [6]. Patients with this mutation typically experience severe CF symptoms when the other allele also carries a CF-causing mutation [7,10,11].

**Figure 1:**
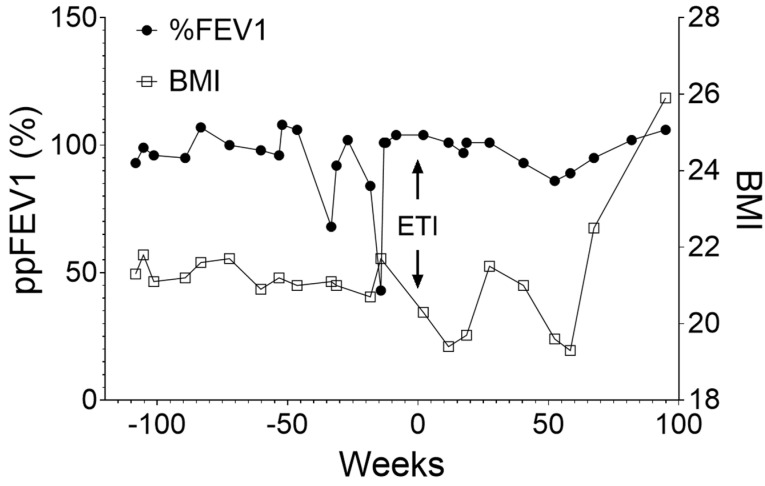
ETI improved the lung function and body development in the patient. Longitudinal observations of percent predicted FEV1 (ppFEV1) and Body Mass Index (BMI), indication of lung function, and body development, respectively, two years before and after ETI initiation. FEV1: Forced Expiratory Volume in one second.

The patient, who receives care at our CF center in Indianapolis, has a long history of CF-related complications, including pancreatic insufficiency, chronic rhinosinusitis, chronic pulmonary infections with pseudomonas and mycobacterium avium complex, chronic abdominal pain, anxiety, and depression. Interestingly, his lung function, as determined by mean ppFEV1, was relatively normal at 99.1% and 89% on average for year 2 and year 1 before ETI, respectively (Table 1 and Figure 1). However, the ppFEV1 has been declining and is unstable. He has also experienced relatively frequent pulmonary exacerbations, requiring three courses of oral antibiotics and two hospital admissions for IV antibiotics. Additionally, he has had a frequent productive cough and intermittent dyspnea with exertion. His mean body mass index (BMI) was around 21.02, showing a declining trend (Table 1 and Figure 1).

### 2.2. In Vitro Therapying Studies

**Figure 2:**
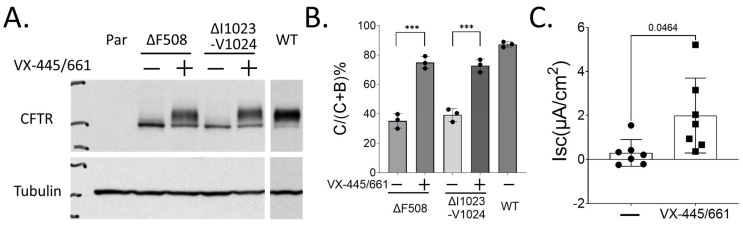
Elexacaftor/Tezacaftor/Ivacaftor improves the expression and function of ΔI1023-V1024-CFTR. (**A**) Representative images of immunoblotting. As described in the Methods Section, ΔF508-, ΔI1023-V1024, and wildtype (WT) CFTR were transiently overexpressed in HEK 293 cells. Cells were then treated with DMSO (−− or VX-445/661 (2 μM) for 48 h before being harvested. Samples were subjected to immunoblotting to probe for CFTR and Tubulin using specific α-CFTR (CFF 596) and α-Tubulin antibodies, respectively. B and C indicate the CFTR immature band and the mature band, respectively. (**B**) The band intensity in (**A**) was quantified using Image Lab v5.0, and the ratio of Band C/(C + B) was determined. Statistical analyses were performed using a Student’s *t*-test in GraphPad Prism 10.3.1. *** represents *p* < 0.001. (**C**) Analysis of CFTR function in response to Forskolin (FSK)/VX-770 in patient-derived nasal epithelial cells (NECs). NECs were cultured on Transwell for 4 weeks to differentiate as described in the Methods Section. Each dot represents one replicate from two independent experiments. Statistical analyses were performed using a Student’s *t*-test in GraphPad Prism 10.3.1. *** represents *p* < 0.001.

In order to understand the defect induced by the mutations, a plasmid expressing ΔI1023_V1024-CFTR was transfected into HEK293 cells. It was found that ΔI1023_V1024-CFTR showed an in vitro response to VX445/661, also known as Elexacaftor/Tezacaftor or ET, respectively. The mature form of ΔI1023_V1024-CFTR (band C) was produced in the presence of CFTR modulators to levels comparable to ΔF508-CFTR upon treatment (Figure 2A,B). This is consistent with previous studies [6]. To assess CFTR function in patient-derived epithelial cells, nasal brushes were obtained, and NECs were isolated and differentiated at an air–liquid interface (ALI) culture as described in the methods. CFTR-dependent chloride transport was determined using a Ussing Chamber assay (Figure S2). It was found that upon treatment with VX compounds for 48 h, CFTR function was marginally restored (Figure 2C), reaching approximately 5% of wildtype CFTR levels.

### 2.3. Improvement in Clinical Outcomes with ETI Treatment

Based on the laboratory research data provided above and following insurance approval, ETI treatment was initiated for the patient in April 2023. We did not observe a statistically significant improvement in lung function (Table 1 and Figure 1). However, lung function, as determined by ppFEV1, has been much more stable in the two years after starting ETI compared to the two years before the initiation of ETI. While the BMI did not change significantly (Figure 1), measurements in year 2, after starting ETI when adherence had improved, indicated a notable improvement in the patient’s recent BMI assessments (Figure 1). It appeared that the number of pulmonary exacerbation episodes was decreasing, and recent sweat chloride measurements tended toward lower chloride values. These observations suggest that ETI was making an impact, although longer-term follow-up is necessary.

### 2.4. Materials and Methods

#### 2.4.1. Ethics Statement

Written informed consent was obtained from the subject according to the IRB 11042.

#### 2.4.2. CFTR Modulators and Chemicals

Elexacaftor (VX-445, cat# HY-111772), Tezacaftor (VX-665, cat# HY-15448), A-83-01 (TGF-β inhibitor), DMH-1 (BMP4 inhibitor), CHIR99021 (Wnt/β-catenin signaling pathway activator), and Y-27632 (ROCK inhibitor) were obtained from MedChemexpress LLC (Monmouth Junction, NJ, USA). Ivacaftor (VX-770, cat# S1144) was acquired from Selleck Chemical LLC (Houston, TX, USA).

#### 2.4.3. Generation of Relevant Plasmids

The template used to generate the plasmid in the study was the wildtype plasmid pcDNA3.1(+)-CFTR. Plasmids expressing ΔF508-CFTR were previously described [2,12]. The plasmid expressing ΔI1023_V1024-CFTR was created using a site-directed mutagenesis kit from Agilent Technologies (Santa Clara, CA, USA) (cat# 200521) with the following primer set: forward CAT CTT TGT TGC AAC AGT GCC AGT GGC TTT TAT TAT GTT GAG AGC ATA TTT C; reverse GAA ATA TGC TCT CAA CAT AAT AAA AGC CAC TGG CAC TGT TGC AAC AAA GAT G. Plasmid isolation from bacteria was conducted using the PlasmidPrep Mini Spin Kit from Cytiva (Marlborough, MA, USA) (cat#: 28904270).

#### 2.4.4. Cell Lysate Preparation and Immunoblotting

HEK293 cells transiently transfected with the indicated plasmids using Lipofectamine 3000 from ThermoFisher (Waltham, MA, USA) (cat# L3000015) were treated with VX-445 and VX-661 for 48 h post transfection. Cells were lysed in IP lysis buffer (ThermoFisher cat# 87787) containing EDTA-free protease inhibitor cocktail (ThermoFisher cat# A32953). Cell debris was removed by centrifugation at maximum speed for 10 min at 4 °C. Samples were subjected to SDS-PAGE followed by immunoblotting. CFTR and tubulin were probed with anti-CFTR (CFF 596 from University of North Carolina, North Carolina, USA) and anti-tubulin from Cell Signaling Technology (Danvers, MA, USA) cat# 2144S) antibodies, respectively. The chemiluminescent signal was captured by the X-ray film, which was scanned using the Bio-Rad (Hercules, CA, USA) ChemiDocTM Touch imaging system and processed using Image Lab (version 6.0).

#### 2.4.5. Nasal Cells Culture

Nasal cell isolation and expansion were previously described [13]. Briefly, a nylon bristle cytology brush was used to brush the inferior turbinate and immediately placed in a 15 mL conical tube containing F medium (75% Ham’s F-12, 25% DMEM, 0.05%FBS, 24 μg/mL Adenine, 8.4 ng/mL Cholera Toxin, 10 ng/mL EGF, 1.105 μM Hydrocortisone, 5 μg/mL Insulin, 10 μM Y-2763, 30 μg/mL Gentamicin, 1xPen/Strep). NECs were scraped off the brush with sterile forceps onto a 10 cm dish and collected into a conical tube. The cells were harvested by centrifugation and treated with trypsin. After neutralization with DMEM/F12 with 10% serum, the cells were centrifuged and resuspended in F media containing Y27632 and Pen/Strep. The cells were then plated on a T25 flask pre-coated with irradiated 3T3 fibroblasts. The media was replaced daily until the cells reached confluency. Subsequently, the cells were harvested and preserved in liquid nitrogen.

Expansion of nasal cells was achieved using the feeder-free “dual-SMAD inhibition” method, as previously described [14,15], with some adaptations. Briefly, a vial of cells was resuspended in expansion medium [LHC-9 medium (ThermoFisher, cat# 12680013) containing 1 μM A-83-01, 0.2 μM DMH-1, 0.5 μM CHIR99021, 5 μM Y27632, Pen/Strep, and 10% FBS] and then plated on a dish pre-coated with 804G-conditional medium. The expansion medium was changed daily until the cells reached confluency. To establish ALI culture, cells were harvested in expansion medium and plated at a density of 165 K/well on a Transwell membrane (6.5 mm) pre-coated with 804G-medium. After an overnight culture, the medium in both the top and bottom chambers was replaced with differentiation medium [PneumaCult-ALI Medium (STEMCELL Technology (Vancouver, BC, Canada) cat# 05050)]. The medium in the top chamber was removed the following day to initiate the ALI environment. Cells were treated with Y27632 and A-83-01 for the first week after plating on the Transwell surface. At week four, the differentiated cells were subjected to Ussing Chamber analysis to determine CFTR function.

#### 2.4.6. Ussing Chambers

Ussing Chambers analysis was performed as previously described [16], using the P2300 EasyMount Ussing System for Cell and Tissue from Physiologic Instruments (San Diego, CA, USA). The system’s temperature was maintained at 37 °C by a circulating water system. The apical side of the cells was bathed in Ringer’s solution [ in mM, 0.12 NaCl, 25 NaHCO_3_, 3.3 KH_2_PO_4_, 0.83 K_2_HPO_4_, 1.2 CaCl_2_, 1.2 MgCl_2_, 141 Na-gluconate, and 10 mannitol, pH 7.2], while the basolateral side was bathed in Ringer’s solution [in mM, 120 NaCl, 25 NaHCO_3_, 3.3 KH_2_PO_4_, 0.83 K_2_HPO_4_, 1.2 CaCl_2_, 1.2 MgCl_2_, and 10 D-glucose, pH 7.2]. The resistance was determined and was larger than 200 Ω across the epithelium layer (Figure S1). After establishing the baseline, the short-circuit current (Isc) was monitored in response to the following treatments at the apical side in the order 25 μM Amiloride, 10 μM FSK, 2 μM VX-770, and 20 μM CFTR_inh-172_. The combined change in Isc following FSK and VX-770 was used to assess CFTR function in the differentiated NECs.

#### 2.4.7. Statistics

The statistical analysis was performed using GraphPad (GraphPad Software, San Diego, CA, USA).

## 3. Discussion

In this study, we report a rare CF-causing CFTR variant, ΔI1023_V1024-CFTR, complexed with an intronic mutation c.1680-877G>T in a 19-year-old male patient. Neither mutation is considered to be responsive to CFTR modulators. We found that ETI could rescue the expression of ΔI1023_V1024-CFTR in a heterologous expression system (Figure 2A) and partially restore CFTR function in the patient-derived NECs (Figure 2C). Since a minigene carrying the c.1680-877G>T mutation could not express CFTR protein at all [8], we expected that the observed CFTR function in NECs was derived from ΔI1023_V1024-CFTR. Importantly, it has been shown that the patient, after taking ETI, showed improvement, implying that treating patients who carry ΔI1023_V1024-CFTR could be supported by ETI therapy.

Current studies utilize two different in vitro models to assess the effect of ETI on ΔI1023_V1024-CFTR. HEK293 cells were used to evaluate its protein expression, while patient-derived NECs were used to determine its function. It was observed that ΔI1023_V1024-CFTR had a similar expression response to ETI as ΔF508-CFTR. In the absence of CFTR modulators, ΔI1023_V1024-CFTR only expressed the immature form (Band B) (Figure 2A). However, in the presence of ETI, a significant portion of ΔI1023_V1024-CFTR was present as the mature form (Band C) (Figure 2B), indicating that ΔI1023_V1024-CFTR exhibited class II mutation characteristics with a trafficking defect. Nevertheless, CFTR function in the patient-derived nasal epithelium carrying ΔI1023_V1024-CFTR could only be marginally rescued (approximately 5% of wildtype, Figure 2C), suggesting that ΔI1023_V1024-CFTR may have a severe gating defect. This finding differs slightly from a previous study in which ΔI1023_V1024-CFTR alone exhibited only a mild gating defect using a membrane depolarization assay in HEK293 cells [6]. It is possible that this discrepancy is due to assay differences, or it could be that the c.1680-877G>T mutation has a negative effect on the ΔI1023_V1024-CFTR function restored by ETI. Given that I1023_V1024-CFTR is located on the 10th transmembrane helix, which is distant from directly regulating the channel gating and interacting with CFTR modulators based on the CFTR structure [17,18], it is speculated that deletion of I1023_V1024 may alter the position of the following intracellular loop 4 (ICL4). This alteration could impair the pairing of E267 on ICL2 and K1060 on ICL4 [19], resulting in a compromised CFTR-conducted Cl^−^ current. However, more studies are needed to investigate this hypothesis.

CFTR modulator theratyping using preclinical models has been suggested as an important approach to support the use of modulator therapies in new genotype-based patient populations [1,2,3]. Among several preclinical model systems, NECs have been suggested as a surrogate for bronchial epithelial cells, which are the gold-standard evaluating CFTR function [3,20]. The ΔI1023_V1024-CFTR variant is rare, accounting for approximately 0.06% of CF-causing variants according to the CFTR2 database. In the current study, we employed the methodology developed by Mou and colleagues using dual-SMAD signaling inhibitors to expand NECs [14]. This culture protocol represents an easier way to expand NECs for research compared to feeder cell-based conditionally reprogrammed culture (CRC) [21]. Several CF studies [22,23] have used this pharmacological compound-based method to expand nasal epithelial stem cells. Although this method can be easily adapted in the laboratory, some concerns have been raised [24] as CFTR shows different functionality using these two methods. Therefore, caution should be exercised in choosing the culture condition for a specific purpose.

In our study, a small but significant increase in CFTR function was observed in response to ETI in the nasal epithelial ALI culture. This response appeared to correlate with the patient’s clinical outcomes after ETI treatment, supporting the use of NECs in predicting clinical response. It should be noted that the patient did not consistently take CFTR modulators initially, but adherence has been improving. Three out of 27 fills were missed over the past two years, two in 2023 and one in 2024. However, there were no missing fills this year. This may explain the delayed clinical response after taking ETI. Our study supports the idea that patients with rare CFTR mutations may benefit from CFTR modulator therapy if a significant increase in CFTR function is evident, even if the response is small. Furthermore, cryo-EM studies investigating ΔI1023_V1024-CFTR structure in the presence or absence of CFTR modulators should be conducted in future research to reveal the molecular mechanisms responsible for the compromised CFTR function.

## 4. Conclusions

Preclinical model systems, particularly patient-derived NECs, are crucial in evaluating the response of emerging CFTR variants in patients to CFTR modulators. CFTR modulator therapy may provide additional benefits to patients with CF whose cells exhibit only a small response to CFTR modulators in vitro.

## Figures and Tables

**Table 1:** Comparison of patient’s clinical measurements before and after ETI.

Mean	y2 Pre-ETI	y1 Pre-ETI	y1 Post-ETI	y2 Post-ETI
ppFEV1 (%)	99.1	89.0	97.6	98.0
Coefficient of variation (ppFEV1)	16.79%		6.49%	
BMI (kg/m^2^)	21.3	21.2	20.4	21.8
Cultures	PA, MSSA			
PEx req IV (n)	1	1	1	0
Sweat Chloride * (mmol/L)	116		108	

Data was collected up to 1/23/205. *: Pre-ETI SwCl was determined 2/24/2009. Post-ETI SwCl was determined 24 October 2024.

## Data Availability

The data presented in this study are available on request from the corresponding authors. The data are not publicly available due to privacy and ethical restrictions.

## Supplementary Materials

The following supporting information can be downloaded at: https://www.mdpi.com/article/10.3390/ijms26115306/s1.

## Abbreviations

ALI: air–liquid interface; BMI: body mass index; CFTR: Cystic Fibrosis transmembrane conductance regulator; ETI: Elexacaftor/Tezacaftor/Ivacaftor; FBS: Fetal Bovine Serum; MSSA: *methicillin-susceptible Staphylococcus aureus*; NECs: nasal epithelial cells; PA: *Pseudomonas aeruginosa*; PEx: pulmonary exacerbation; ppFEV1: percent predicted forced expiratory volume in 1 s.
