# Supplementary material for: Elexacaftor/Tezacaftor/Ivacaftor Supports Treatment for CF with ΔI1023-V1024-CFTR
Source: Int J Mol Sci. 2025 May 31;26(11):5306. doi: 10.3390/ijms26115306 (PMC12155120; doi:10.3390/ijms26115306)
Supplement: Supplementary file 1 [file ijms-26-05306-s001.zip › ijms-3642647-supplementary.pptx]

## Slide 1
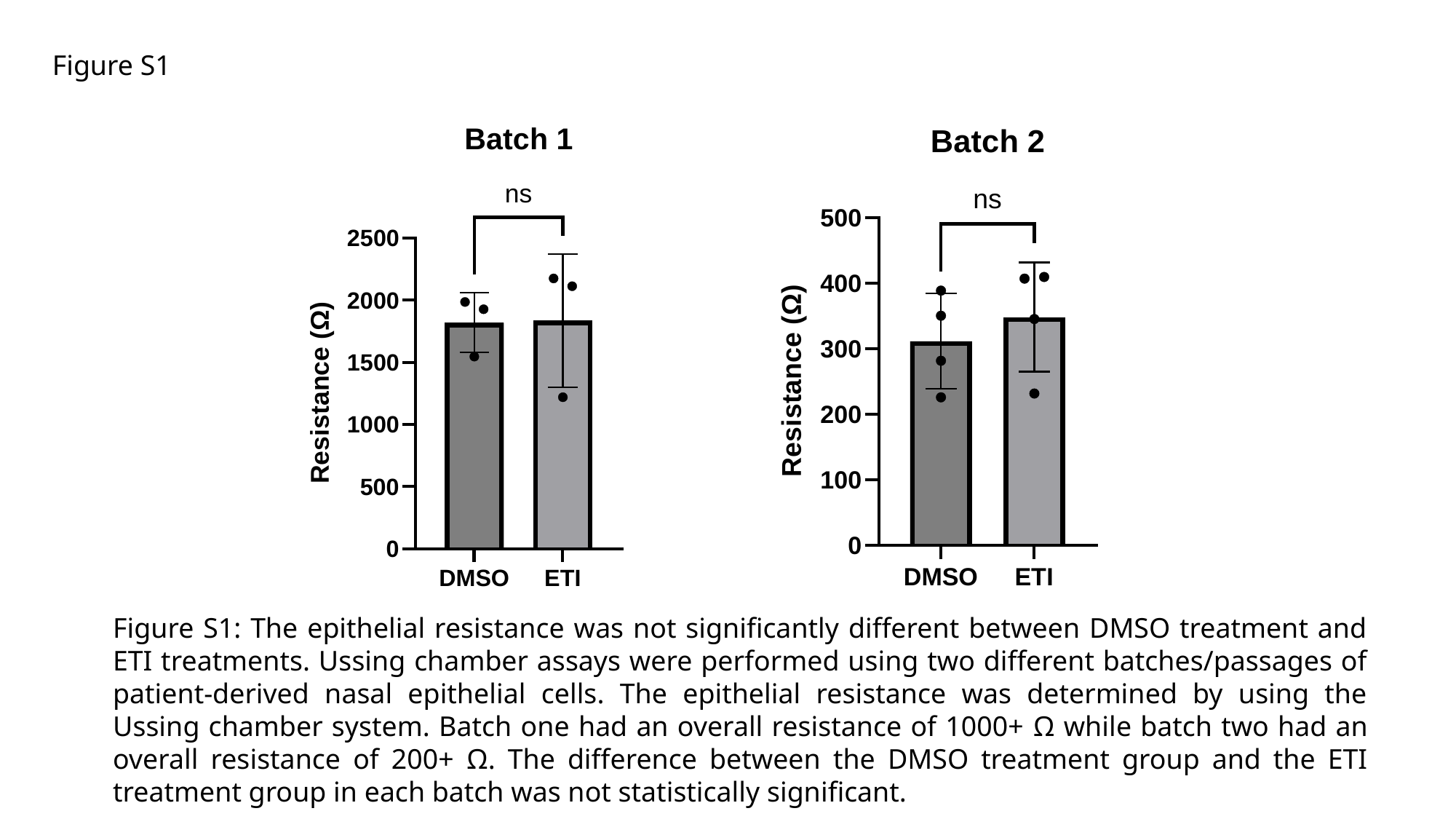

Figure S1
Figure S1: The epithelial resistance was not significantly different between DMSO treatment and ETI treatments. Ussing chamber assays were performed using two different batches/passages of patient-derived nasal epithelial cells. The epithelial resistance was determined by using the Ussing chamber system. Batch one had an overall resistance of 1000+ Ω while batch two had an overall resistance of 200+ Ω. The difference between the DMSO treatment group and the ETI treatment group in each batch was not statistically significant.

## Slide 2
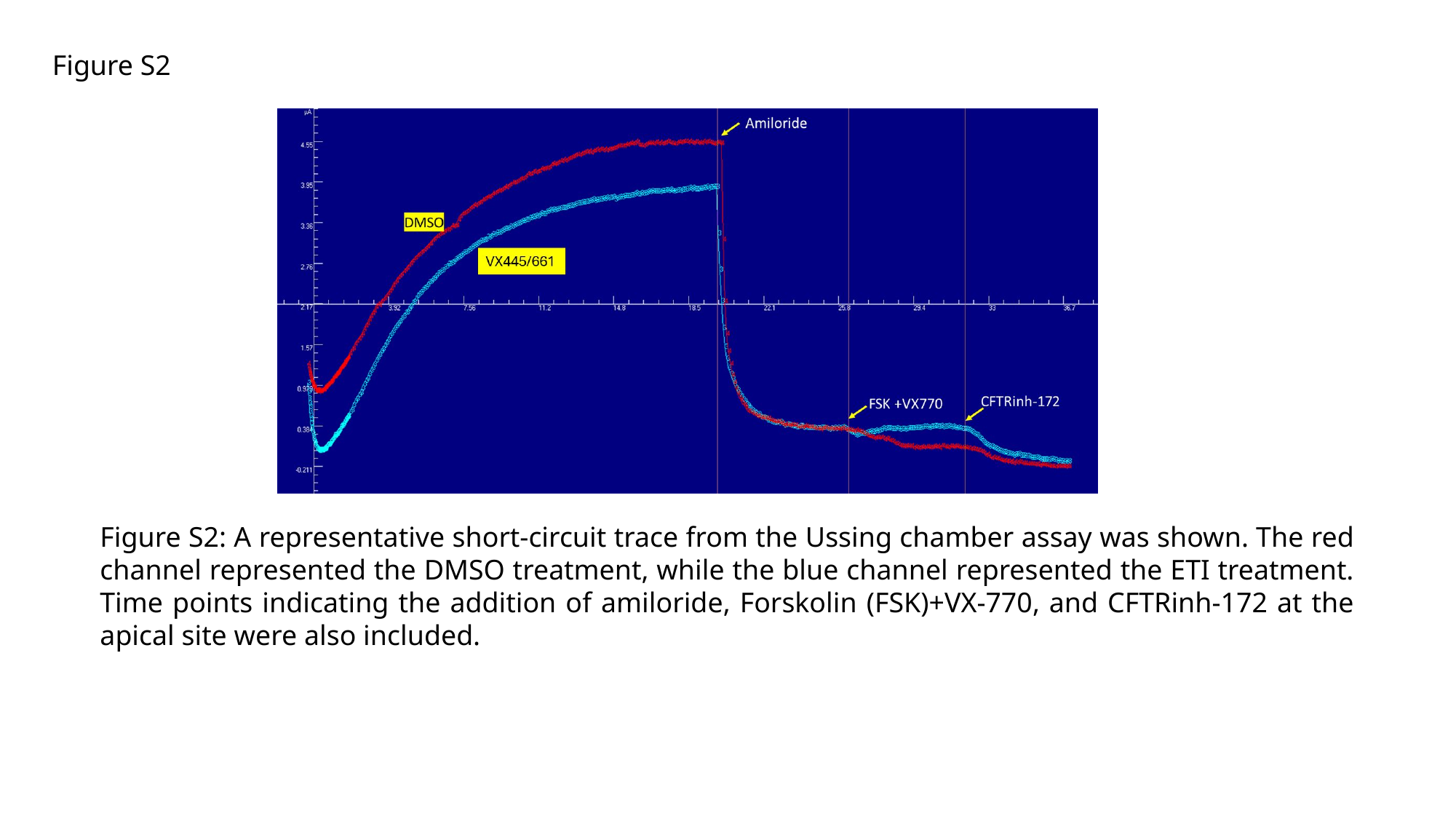

Figure S2
Figure S2: A representative short-circuit trace from the Ussing chamber assay was shown. The red channel represented the DMSO treatment, while the blue channel represented the ETI treatment. Time points indicating the addition of amiloride, Forskolin (FSK)+VX-770, and CFTRinh-172 at the apical site were also included.
